# Cytotoxic and mutagenic potential of solutions exposed to cold atmospheric plasma

**DOI:** 10.1038/srep21464

**Published:** 2016-02-24

**Authors:** Daniela Boehm, Caitlin Heslin, Patrick J. Cullen, Paula Bourke

**Affiliations:** 1 Plasma Research Group, College of Science and Health, Dublin Institute of Technology, Dublin 1, Ireland; 2 School of Chemical Engineering, University of New South Wales, Sydney, Australia

## Abstract

The exposure of aqueous solutions to atmospheric plasmas results in the generation of relatively long-lived secondary products such as hydrogen peroxide which are biologically active and have demonstrated anti-microbial and cytotoxic activity. The use of plasma-activated solutions in applications such as microbial decontamination or anti-cancer treatments requires not only adequate performance on target cells but also a safe operating window regarding the impact on surrounding tissues. Furthermore the generation of plasma-activated fluids needs to be considered as a by-stander effect of subjecting tissue to plasma discharges. Cytotoxicity and mutagenicity assays using mammalian cell lines were used to elucidate the effects of solutions treated with di-electric barrier discharge atmospheric cold plasma. Plasma-treated PBS inhibited cell growth in a treatment time-dependent manner showing a linear correlation to the solutions’ peroxide concentration which remained stable over several weeks. Plasma-treated foetal bovine serum (FBS) acting as a model for complex bio-fluids showed not only cytotoxic effects but also exhibited increased mutagenic potential as determined using the mammalian HPRT assay. Further studies are warranted to determine the nature, causes and effects of the cyto- and genotoxic potential of solutions exposed to plasma discharges to ensure long-term safety of novel plasma applications in medicine and healthcare.

Non-thermal plasma, generated by the ionisation of gases, consists of free electrons, radicals, positive and negative charged particles has shown strong effects on living cells. Plasma can cause both cell death and stimulate cell proliferation and has found applications in microbial decontamination, wound healing and cancer treatment^1^,^2^,^3^,^4^. The inactivation of bacteria and fungi by cold plasma, and similar cytotoxic effects on eukaryotic cells have been suggested to result predominantly from oxidative damage to the cells’ membrane and intra-cellular components including DNA through the action of reactive oxygen species (ROS)^5^,^6^.

A number of recent publications have reported on the biological activity of plasma treated solutions such as plasma-activated water (PAW), plasma-activated PBS (PAPBS) or plasma-activated medium (PAM) which were found to possess anti-microbial and/or cytotoxic activity^7^,^8^,^9^. These solutions are of interest as novel anti-microbial agents for decontamination of surfaces, wounds and food products^7^,^8^,^10^ and may offer similar antimicrobial effects as observed with direct exposure to the plasma discharge. The retention of efficacy in solution would provide the advantages of off-site production, storability and ease of application over direct treatment. Plasma-activated medium has furthermore shown selective cytotoxicity in several cancerous cell lines, including chemotherapy resistant types, making it a novel candidate treatment for anti-cancer therapies^9^,^11^,^12^. Atmospheric cold plasmas which may be generated by a range of different plasma devices such as plasma jets, di-electric barrier discharge, corona discharge or gliding arc contain an array of biologically relevant reactive oxygen and nitrogen species such as ozone, atomic oxygen, superoxide anion, hydroxyl radical, hydrogen peroxide, nitric oxide and peroxynitrite^13^. The exposure of aqueous solutions to atmospheric plasmas results in the generation of relatively long-lived secondary products such as hydrogen peroxide, nitrates and nitrites which may react to form further bactericidal compounds such as peroxynitrous acid^7^,^14^. Hydrogen peroxide has been implicated as a key factor in the cytotoxic and DNA damaging effects of extended plasma treatment on mammalian cells while ozone concentrations were suggested to play a minor role when studied in a keratinocyte cell line^15^. Comparing the effects of ozone to air and helium plasmas, Lunov *et al.* revealed differences in activation of signalling cascades between the plasma types and found both faster and higher cytotoxic effects of ozone on fibroblast cells but did not show if similar amounts of ozone were produced in the plasma discharge^16^.

Sato and co-workers compared the effect of plasma-treated cell culture medium and H_2_O_2_ supplemented cell culture medium on growth, viability and gene expression profiles of HeLa cells and found them to be largely comparable. Pre-treatment with catalase prevented cytotoxic effects induced by either type of medium with the authors suggesting H_2_O_2_ to be the main stable chemical species responsible for cytotoxicity^17^. H_2_O_2_ in liquids has been suggested to result predominantly from the solubilisation of H_2_O_2_ formed under plasma in the gaseous phase and is dependent on the humidity of the gas phase while a smaller amount of H_2_O_2_ is formed directly by the action of plasma on the liquid^18^. Xu *et al.* recently proposed a mechanism whereby H_2_O_2_ and O_2_^−^ are the main reactive species that are produced by plasma treatment and the generation of OH radicals within the cells via the Haber-Weiss reaction, resulting in apoptosis and cell death^19^.

However, other studies have observed differences in cell viability with varying nitrogen to oxygen ratios which is attributed to the effect of other cytotoxic reactive species such as nitrites or nitrates, when H_2_O_2_ concentrations remained unchanged^18^,^20^.

While the retention of biologically active compounds in plasma-treated solutions opens up a range of new fields of application, this approach also needs to be evaluated in terms of safety implications and considered as a potential hazardous bystander effect of direct plasma treatment where liquid is present such as the bio-fluids in a wound setting.

This study focussed on the cytotoxic effects of dielectric barrier discharge cold atmospheric plasma and their dependency on the surrounding medium. Hydrogen peroxide generation as one of the main effector molecules produced in aqueous solution during plasma discharge in air is correlated to the observed cytotoxic effects. The potential of high-voltage plasma exposure to induce mutagenic effects in plasma treated solutions - both simple buffered saline solutions and complex bio-fluids - was assessed and compared to supplementation with hydrogen peroxide.

## Results

### Cytotoxicity of plasma treatment is dependent on the extra-cellular milieu and is retained post-exposure

**Figure 1:**
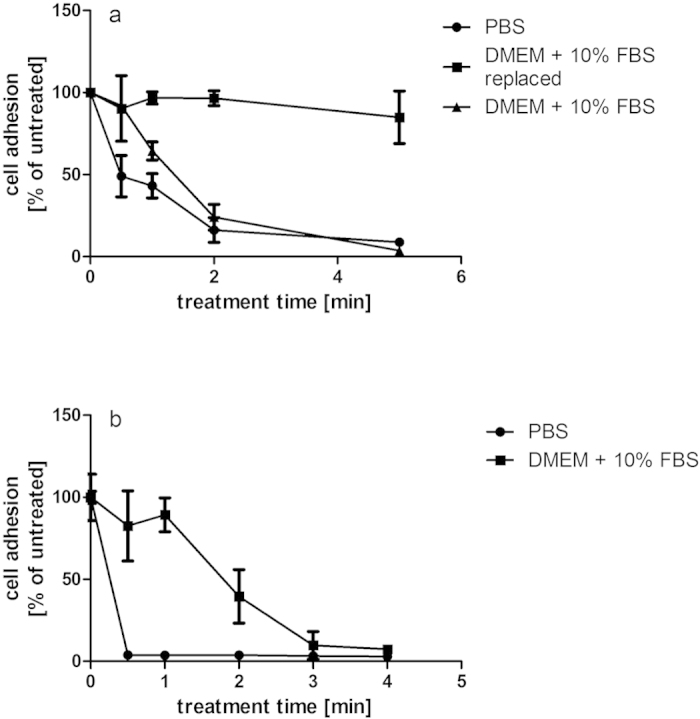
(**a**,**b**) Cell re-adhesion and growth after plasma treatment at 70 kV_RMS_ are dependent on treatment times and medium. Cells were seeded at 1 × 10^6 ^cells/ml and cell adhesion assessed 1 day post treatment (**a**) or cells were seeded at 2.5 × 10^5 ^cells/ml and cell adhesion was assessed 3 days post-treatment (**b**) using crystal violet staining.

**Figure 2:**
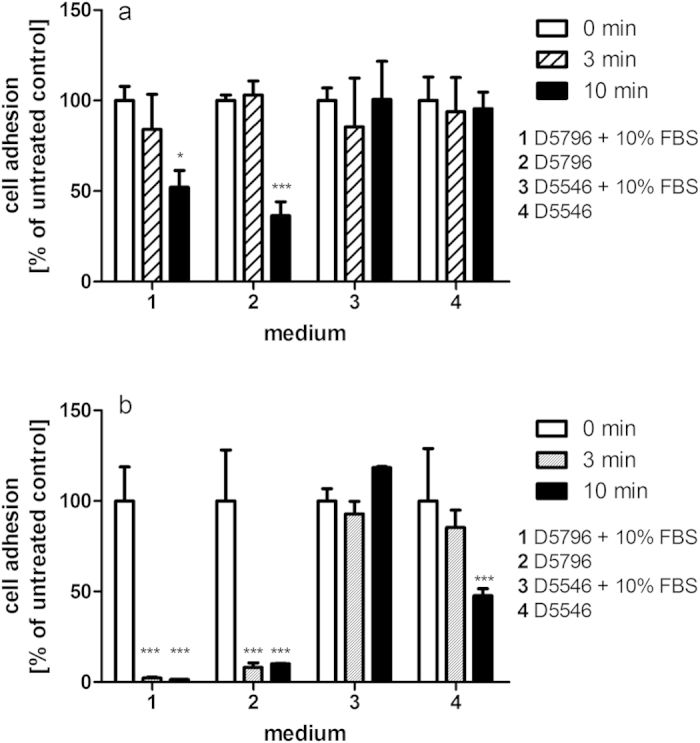
(**a**,**b**) Cytotoxic effects of medium treated with DBD-ACP at 70 kV_RMS_ are dependent on treatment time and medium formulation as seen for cell re-adhesion (**a**) and cell growth (**b**). Asterisk indicates significant difference to control; *p < 0.05; **p < 0.01; ***p < 0.001.

**Figure 3:**
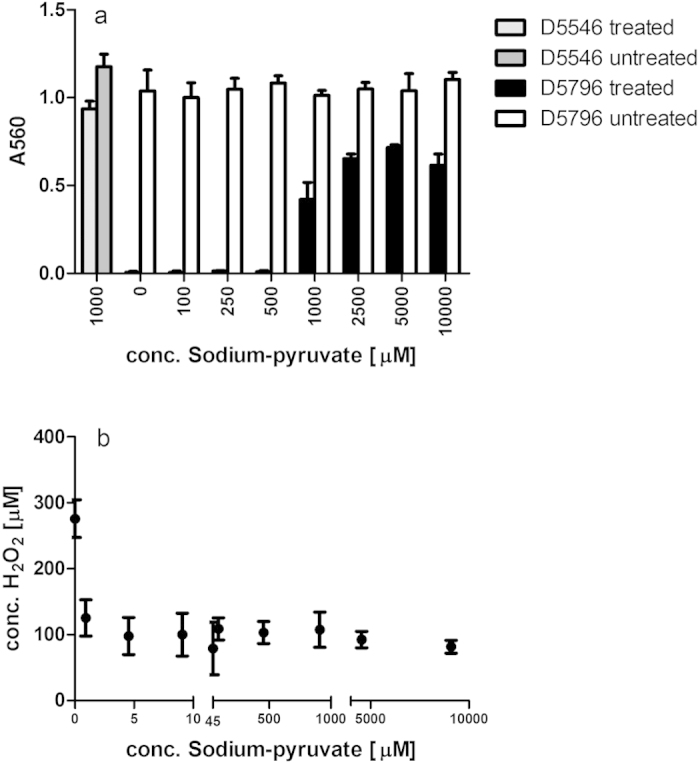
(**a**,**b**) Supplementation with pyruvate can reduce the cytotoxic effects observed in plasma-treated DMEM medium D5796 as assessed using the crystal violet assay (**a**) and reduces the amount of hydrogen peroxide generated in solution by plasma exposure (**b**).

Cytotoxic effects of plasma treatment on HeLa cells were found to be strongly dependent on both the medium in which the cells were treated (protective effects) and the change of medium after treatment (retention or removal of cytotoxic effects). Results obtained using crystal violet staining were confirmed by the MTT assay indicating reduced metabolic activity of these cells (results not shown). Treatment of detached cells in phosphate buffered saline (PBS) resulted in an impaired ability of cells to re-adhere to surfaces after treatment for longer than 30 seconds (Fig. 1a) and strongly reduced cell growth (Fig. 1b). Cells treated in normal growth medium DMEM supplemented with 10% foetal bovine serum (FBS) showed much lower sensitivity to plasma treatment, where the effects were reversible by replacement of the medium after treatment (Fig. 1a,b), suggesting a retention of cytotoxic effects in the medium to have a stronger impact on the cells. Haertel *et al.* also found the immediate replacement of medium to reduce cytotoxic effects on adherent HaCaT cells, but cell recovery in their system still remained significantly below controls at treatments above 40 sec^21^. In agreement with reported effects of ‘plasma-activated medium’^11^,^12^,^18^,^21^,^22^ , cytotoxic effects were also detected when cells were not treated directly but cultured in medium subjected to plasma exposure (Fig. 2). Overall, continuous cell growth (Fig. 2b) was more severely impacted than the short-term re-attachment of cells (Fig. 2a). Cytotoxic effects were dependent on the medium composition with serum (FBS) supplementation providing protective effects and strong differences were observed between different formulations of even the same cell culture medium (DMEM), where DMEM D5796 containing 4.5 g/l glucose showed much higher cytotoxic effects than DMEM D5546 with 1.0 g/l glucose and containing pyruvate at 0.11 g/l. The presence of pyruvate was primarily responsible for this difference as has been observed by others^9^. Supplementation with pyruvate was able to revert most of the growth reduction (Fig. 3a) and reduced the generation of H_2_O_2_ in the plasma-treated solution (Fig. 3b). Of note, these findings highlight the need for standardization of experimental conditions as has been emphasized by others and shows that specification of experimental conditions needs to even exceed beyond product name and supplier details in order to ensure comparability and reproducibility in developing applications where interaction with the extracellular milieu is inherent.

### Complex bio-fluids retain cytotoxic effects after plasma treatment

**Figure 4:**
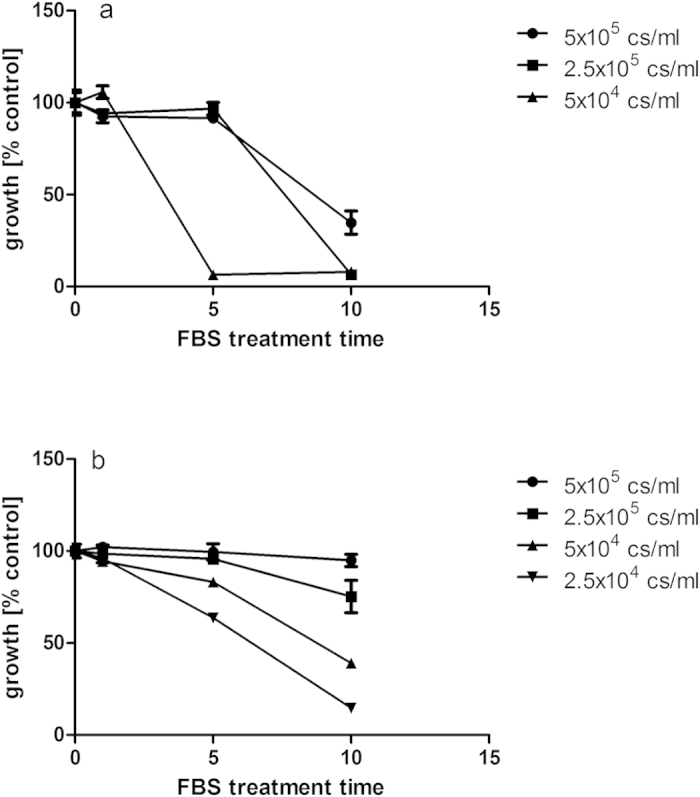
(**a**,**b**) Growth inhibition of plasma-treated FBS on HeLa (**a**) and CHO-K1 cells (**b**) is dependent on plasma exposure time. FBS was treated at 80 kV_RMS_ and stored for 24 hours post treatment. Cells were seeded between 2.5 × 10^4^ and 5 × 10^5 ^cells/ml.

Cells cultured in plasma-treated medium or maintained in plasma-exposed medium after treatment demonstrated a strong inhibition of cell growth, indicating that cytotoxic activities persisted in these solutions in line with reports of plasma-activated media and such effects were reduced by the presence of FBS (Figs 1 and 2) as has been reported by others^22^. Foetal bovine serum (FBS) was subsequently used as a model system representing a complex bio-fluid, containing a range of different lipids, proteins and carbohydrates. Commonly used as a cell culture supplement at 10% v/v, it provided a simple means of assessing the generation of potentially cytotoxic components in bio-fluids upon plasma exposure. FBS treated with plasma for 5 or 10 min caused strong growth inhibition on HeLa cells with effects more strongly visible at lower cell concentrations (Fig. 4a). At high cell concentrations, cells are near confluency after re-attachment and growth is limited by the lack of available surface area, thus causing cell growth to reach a plateau which may mask some the effects of plasma-treated FBS on cell growth. At very low cell concentrations cells showed a much stronger response to the addition of the treated serum. Adachi *et al.* found the cytotoxicity of plasma activated medium to decrease with increasing cell density and correlated this effect with the ability of cellular catalase to detoxify H_2_O_2_^9^.

**Figure 5:**
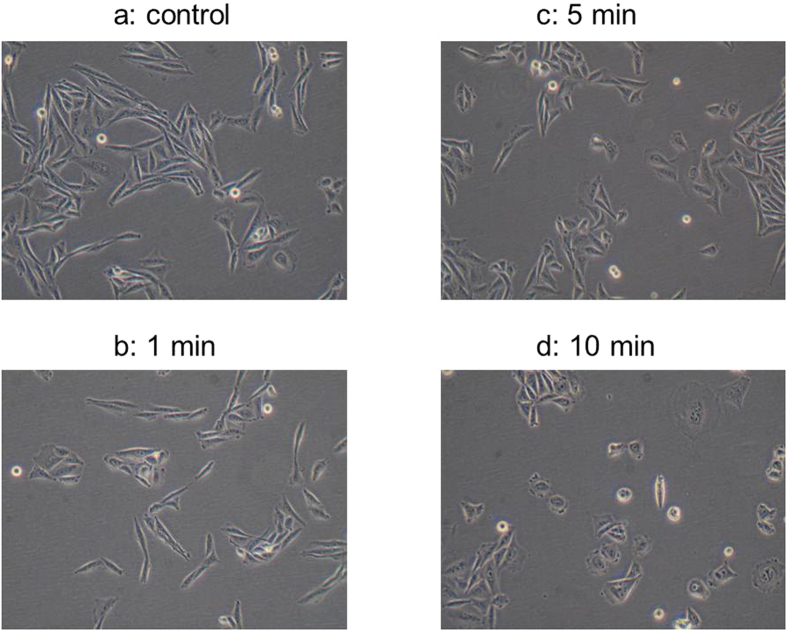
Morphological changes in CHO-K1 cells in response to supplementation with plasma-treated FBS at 10% (v/v).

Growth reduction showed a near linear correlation to treatment time at low cell concentrations in the hamster cell line CHO-K1 (Fig. 4b) which was subsequently used to assess mutagenic effects using the HPRT assay. Plasma treated FBS stored under refrigerated conditions for more than 30 days after opening of the package still caused growth inhibition. This suggests that factors responsible for the inhibition were stable over time. The cellular morphology of CHO-K1 cells was affected by cultivation with plasma treated FBS, displaying increased round cell morphology compared to the normal extended fibroblast shape (Fig. 5). The change was most strongly visible in cultures containing FBS treated for 10 min (Fig. 5d).

**Figure 6:**
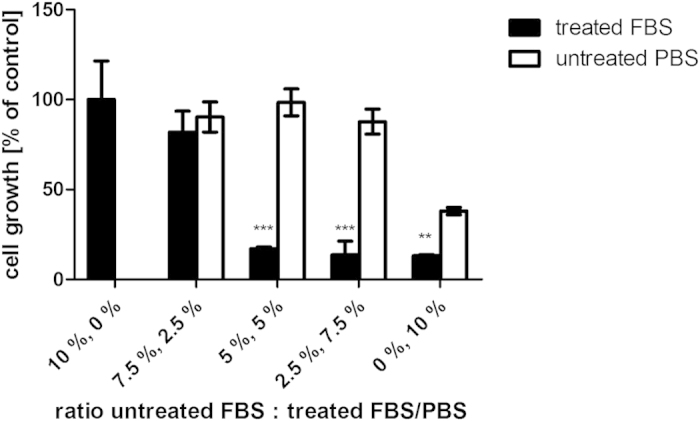
Cell growth in response to supplementation of medium with FBS treated with plasma at 80 kV_RMS_ for 10 min or respective amounts of untreated PBS. Asterisk indicates significant difference between PBS and FBS supplementation; *p < 0.05; **p < 0.01; ***p < 0.001.

Exposure to plasma discharge has been found to result in chemical modifications to various molecules including proteins, amino acids or lipids^6^,^23^,^24^,^25^. To determine whether the growth reduction was a result of the destruction of beneficial factors in FBS which are essential for cell growth (growth factors, hormones), e.g. through plasma-induced changes in protein structure of these factors, or if the growth reduction was associated with the generation of harmful compounds, different volume fractions of FBS were substituted either by treated FBS or by a simple buffered solution (PBS). In the case of a destruction of beneficial factors, a substitution with PBS should show the same degree of growth inhibition as observed for plasma-treated FBS, whereas no such inhibition by PBS would be observed if the reduction in cell growth resulted from inhibitory factors which were formed in the plasma-treated FBS (Fig. 6).

For supplementation of cells with concentrations of 0–10% of untreated PBS or treated FBS, respectively, where the balance was made up of untreated FBS, it could be observed that strong growth inhibition occurred at treated FBS concentrations above 2.5% (Fig. 6). No such inhibition was visible for cultures substituted with the corresponding amounts of PBS (Fig. 6). Reduced cell growth was only found in cultures devoid of any FBS (i.e. 10% PBS, 0% FBS), due to the dependence of the cells on growth factors contained in the serum. Thus, it can be concluded that high voltage plasma treatment inhibited cell growth not due to a destruction of factors vital to cell proliferation but actively generated growth-inhibiting factors or reactive species in the FBS.

### Simple buffered solutions retain cytotoxic effects after plasma treatment

As demonstrated in Fig. 1, direct exposure of mammalian cells to plasma treatment in a simple buffered solution showed the most severe cytotoxic effects due to a lack of antioxidant compounds which are present in the medium. Subsequently, the potential of buffered solutions to retain cytotoxic activity was investigated.

**Figure 7:**
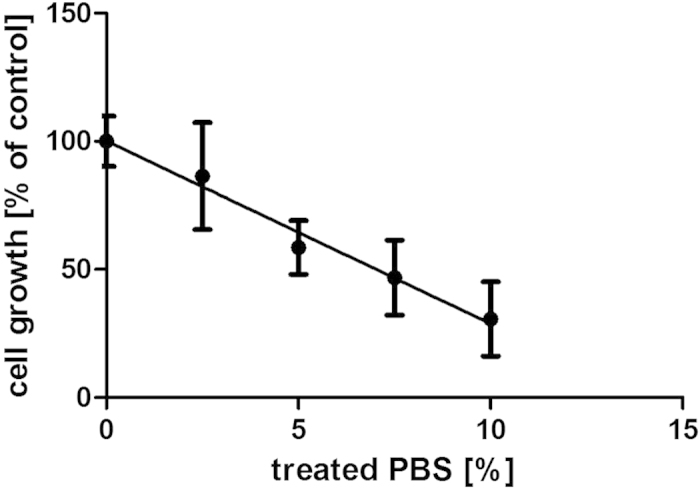
Cell growth in response to supplementation with PBS treated with plasma at 80 kV_RMS_ for 10 min. Growth inhibition by treated PBS shows a linear correlation to the percentage of treated PBS (treated PBS was supplemented with untreated PBS to a total of 10% of the total culture volume).

PBS solution was exposed to di-electric barrier-discharge at 80 kV_RMS_ for 10 min and stored for 24 hours post-treatment. Cells supplemented with treated PBS between 0 and 10% of total volume showed a concentration-dependent reduction of cell growth (Fig. 7). This cytotoxic effect was retained in PBS over extended storage at 4 °C and suggests the formation of stable cytotoxic compounds.

### Hydrogen peroxide concentrations indicate cytotoxic effects

Exposure to plasma discharges has been found to result in the generation of large amounts of hydrogen peroxide in aqueous solutions which are likely to account for a substantial degree of the observed cytotoxic effects on mammalian cells. The cytotoxic effect of hydrogen peroxide has been demonstrated in a range of different cell lines and is believed to involve the *in vivo* generation of OH radicals through the Fenton reaction resulting in DNA strand breaks and damage to other cellular components when produced in close proximity to these^26^,^27^.

**Figure 8:**
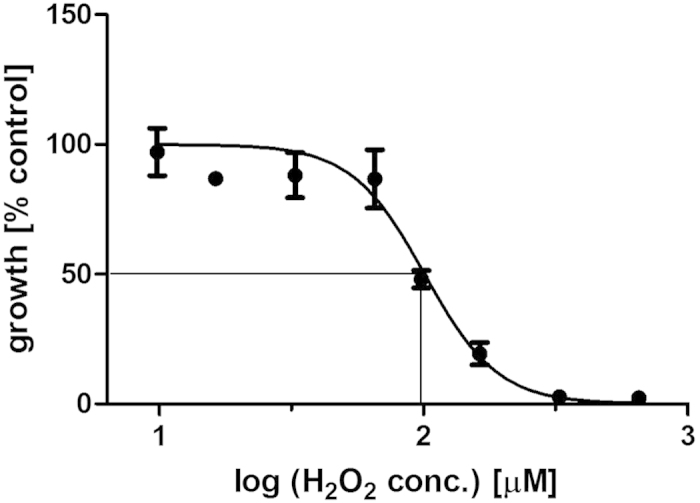
Dose-response curve for treatment of CHO-K1 cells with H_2_O_2_.

**Figure 9:**
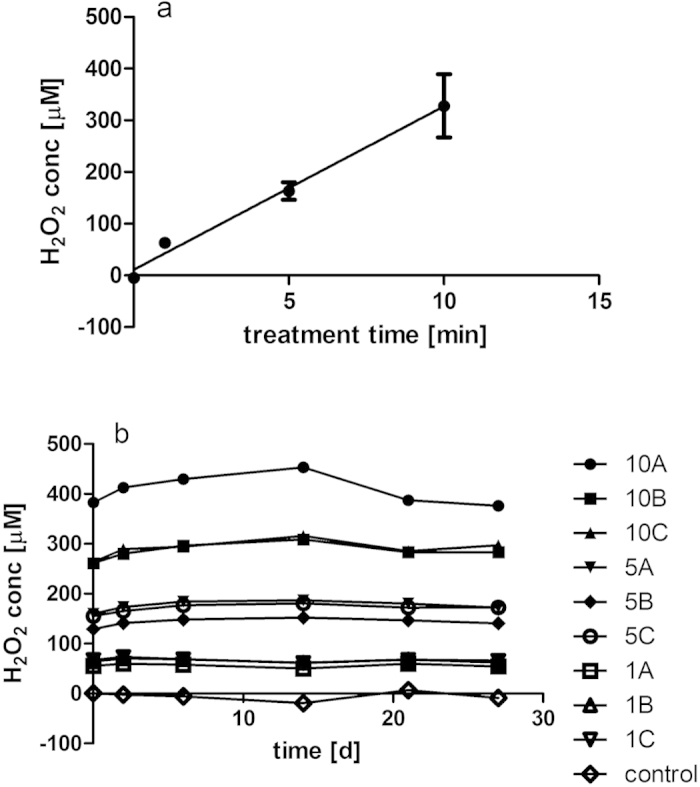
(**a**,**b**) Peroxide concentrations in plasma-treated PBS depend on treatment time (**a**) and remain stable over extended storage periods (**b**). PBS was treated at 80 kV_RMS_ for 1, 5 or 10 min and stored for 24 hours at room temperature in a sealed container, A–C indicate independently treated samples.

H_2_O_2_ concentrations above 100 μM were found to be severely cytotoxic to the mammalian CHO-K1 cells used here (Fig. 8) and are in line with IC_50_ values of 140 μM which have been reported for the cell line^28^. Peroxide levels in treated PBS were determined to be in the range of 50–400 μM H_2_O_2_ depending on treatment duration in conjunction with a 24 hour post-treatment storage time in a sealed container (Fig. 9a). A majority of H_2_O_2_ in plasma-treated solutions has been reported to result from its solubilisation from the gaseous phase^18^, thus by retaining reactive species in the gas phase in contact with the treated liquid for extended periods of time, higher amounts of H_2_O_2_ were able to enter into solution. The concentrations of peroxides in PBS solutions are found to be stable over an extended storage time of several weeks in a closed container at 4 °C (Fig. 9b).

**Figure 10:**
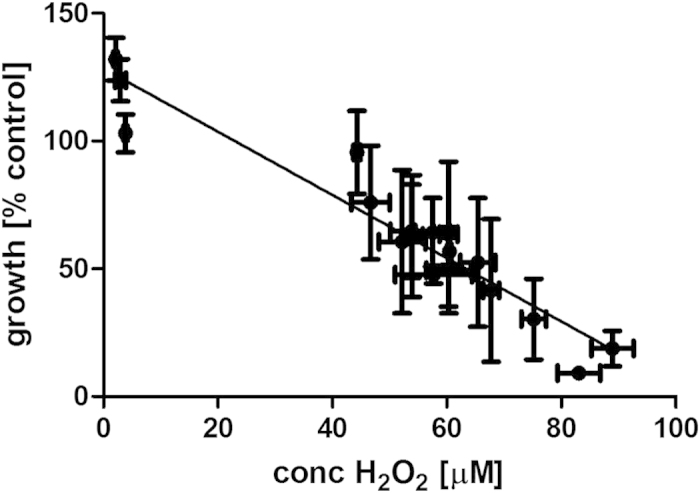
The cytotoxic effect of plasma-treated PBS shows linear correlation to the concentrations of peroxide determined in these solutions.

**Figure 11:**
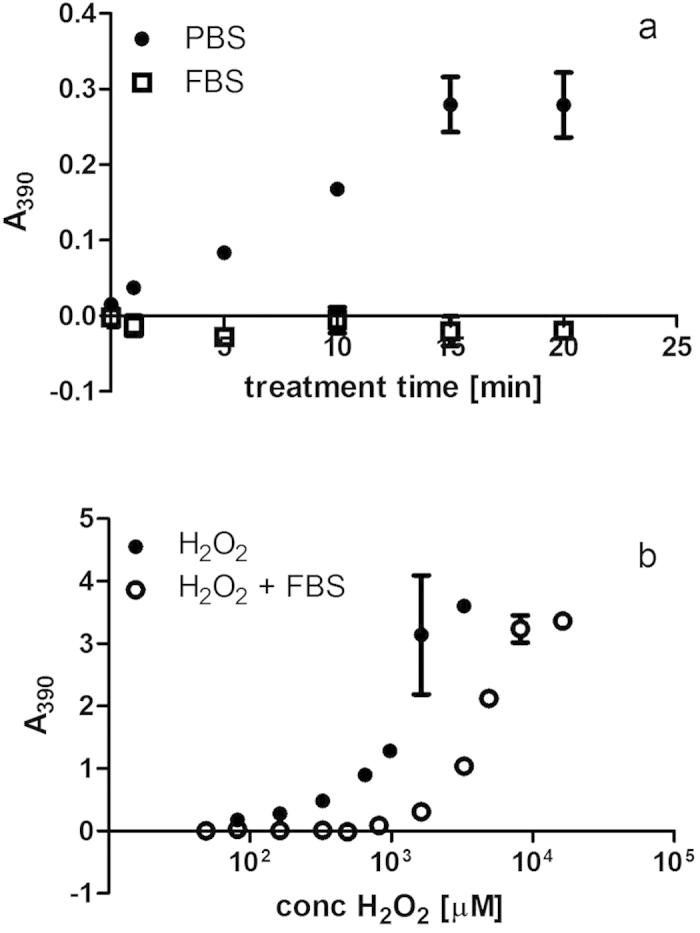
(**a**,**b**) Foetal bovine serum (FBS) acts as scavenger for hydrogen peroxide in plasma treated solution compared to treated PBS (**a**) and neutralizes concentrations below 1000 μM hydrogen peroxide in titration experiments (**b**). Hydrogen peroxide detection was based on the oxidation of KI to iodine and spectrophotometric measurement at 390 nm. Plasma treatment was performed at 80 kV for 0–20 min. Titration was performed at a 1:1 ratio of FBS and commercial hydrogen peroxide at various concentrations.

Increasing exposure times of PBS to the plasma discharge was found to reduce cell growth and showed a linear correlation to the concentration of peroxide generated in the solution (Fig. 10). Peroxide measurements therefore present a very simple and rapid means of estimating the cytotoxic effect of simple DBD-plasma-treated solutions on mammalian cells and may be used as a predictive tool for determining treatment parameters. In contrast, plasma-treated FBS contained no detectable hydrogen peroxide concentrations with increasing plasma treatment times, reflecting the ability of this complex solution consisting of proteins, carbohydrates, amino acids and lipids to scavenge large amounts of H_2_O_2_ (Fig. 11a). As titration experiments showed that FBS can neutralize up to 1000 μM H_2_O_2_ (Fig. 11b), hydrogen peroxide contained in treated FBS was therefore not the main effector of cytotoxicity here and suggests that the generated hydrogen peroxide had reacted with other components, possibly generating secondary reactive species. The strong cytotoxic activities detected in FBS in the absence of detectable levels of H_2_O_2_ indicate that exposure to plasma /plasma reactive species induced changes to the composition of the serum (such as lipid peroxidation, oxidation of proteins, amino acids) which are cytotoxic.

### Plasma-activated solutions possess mutagenic potential in long-term exposure *in vitro* studies

**Figure 12:**
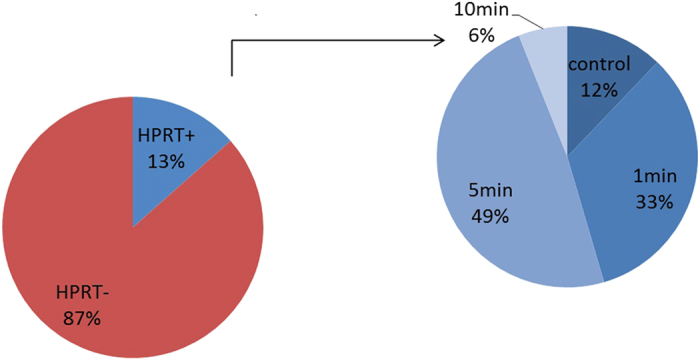
Total occurrence of HPRT+ colonies in cultures supplemented with FBS exposed to DBD-ACP for 0, 1, 5 or 10 min.

While serum solutions contain a range of molecules that can scavenge reactive species, they are also rich in lipids which can generate toxic by-products such as Malone dialdehyde (MDA) or 4-hydroxynonenal (4-HNE) through lipid peroxidation reactions. These molecules have been found to be mutagenic through the formation of DNA-adducts^29^,^30^. The potential of plasma-activated FBS to induce mutations at the *hprt* locus in CHO-K1 cells exposed to this solution over an extended cultivation period was therefore determined. Over a 39 day cultivation period CHO-K1 cells cultured with PA-FBS treated for 5 min, showed mutant colonies on 6 out of 9 sample days whereas control cultures were positive for colony formation on only 2 out of 9 days (Table 1). Of 245 total plates which were assessed for colony formation, 13% were positive. Of the positives, 5 min treated FBS accounted for 49%, 1 min treated for 33%, control for 12% and 10 min for 6%, indicating an increase in mutagenic events in cultures with treated FBS (Fig. 12). The low occurrence of mutagenic events in 10 min treated FBS could be accredited to the significantly higher cytotoxic effects of this medium which reduces the overall viable cell concentration. Hydrogen peroxide itself has been reported as a potential mutagen due to its ability to induce DNA single and double-strand breaks and has shown mutagenic activity in HPRT assays using T-lymphocytes^31^. In a direct comparison of plasma-treated PBS and FBS, both of these solutions exhibited higher occurrence rates of mutations than the untreated controls (Tables 2 and 3). Cultures supplemented with hydrogen peroxide concentrations close to the IC_50_ of 100 μM and within the range of concentrations detected in solution after plasma treatment also showed increased occurrence of HPRT+ cells (Table 4). In light of the presence of substantial amounts of hydrogen peroxide in plasma-treated solutions, a long-term genotoxic effect is not only possible but is likely.

## Discussion

Non-thermal plasmas have been receiving increasing attention for their potential to disinfect biomaterials with applications in surface sterilization or in-package treatment of food products and medical devices. More recently an emerging field of biomedical applications of cold plasmas including blood coagulation, dentistry, cancer treatment and wound decontamination/healing has emerged in what has been termed ‘plasma medicine’.

Plasma-activated water (PAW) or aqueous solutions are furthermore emerging as novel anti-microbial solutions^8^ having been shown to cause cell membrane damage and cell death^5^. Investigations of the chemical composition of PAW and plasma-activated PBS have shown a strong decrease of the solution’s pH, increases in oxidation/reduction potential (ORP) and conductivity^10^ and the generation of H_2_O_2_, nitric acid and nitrous acid^14^,^32^. Plasma-activated media, containing hydrogen peroxide and nitrites/nitrates, has furthermore been reported to initiate the apoptotic cascade in mammalian cells with selectively higher cytotoxicity in cancerous cell lines^9^,^11^. Using a high-voltage DBD system we found H_2_O_2_ concentrations increased with plasma treatment duration in buffered saline solution and that these concentrations remained stable over extended storage periods. These PBS solutions showed strong cytotoxic effects on both HeLa and CHO-K1 cells which correlated with the peroxide concentrations in solution. While H_2_O_2_ is a known cytotoxic agent and has been reported as one of the main mediators of cold plasma cytotoxicity^9^,^18^,^33^, cell growth was inhibited at concentrations lower than those in cultures supplemented with commercially available H_2_O_2_. In agreement with observations made by other studies^14^,^18^,^20^, it is thus likely that peroxide is not the only toxic species present in the plasma-activated solutions obtained by DBD-ACP discharge here, but that other cytotoxic components increased with plasma exposure time in line with H_2_O_2_. The formation of nitrogen-oxide products such as peroxinitrous acid or peroxinitrite from hydrogen peroxide and nitrates/nitrites has been proposed to be the cause of synergistic anti-microbial effects of these compounds and may also account for enhanced cytotoxicity^7^,^14^. Experimental evidence suggests that plasma reactive species lead to an increase in intracellular reactive oxygen species which can stimulate cell proliferation or cytokine release at low concentrations but can also cause DNA damage, induce cell cycle arrest and trigger apoptosis at higher concentrations^34^,^35^,^36^,^37^. While the induction of apoptosis through the generation of intracellular ROS has been postulated as the pathway through which non-thermal plasmas may be a promising therapy for tumour treatment^35^,^38^,^39^,^40^, it also bears the risks of causing oxidative stress-induced genetic and epigenetic alterations involved in carcinogenesis^41^.

In agreement with results reported by others^22^ serum showed strong protective effects in the direct plasma treatment of mammalian cells presumably through scavenging of reactive species such as H_2_O_2_ which have harmful effects on cells. Yan *et al.* have described the possibility of modulating the toxic effects of plasma stimulated medium on glioblastoma cells through the addition of protective FBS, where they observed a consumption of reactive nitrogen species^22^. At the same time, however, we found treated serum solutions themselves to retain cytotoxic activity after prolonged plasma exposure, therefore suggesting the generation of more stable compounds such as lipid peroxides. Lipid peroxidation of cell membranes has been suggested as a major contributor to cellular damage caused by plasma exposure^25^. However, peroxidation products such as malone dialdehyde (MDA) or 4-hydroxynonenal (4-HNE) are also highly reactive species themselves which can form covalent adducts with proteins and DNA and thus cause cell damage^42^,^43^. Due to their relative stability these products need to be considered as toxic and potential mutagenic mediators retained in lipid-containing plasma-treated solutions^29^.

Investigations on the potential genotoxicity of plasma treatments and plasma-treated solutions are still relatively limited and complicated by the vast array of different plasma devices and treatment regimens which are not directly comparable.

Kalghatgi *et al.* analysed DNA damage in MCF10A cells by determining phosphorylation of histone H2AX after exposure to DBD directly or to medium exposed to the discharge and found that neutral species produced by plasma in gas phase initiated this damage via the generation of intracellular ROS^6^. However, the authors did not detect any DNA damage resulting from plasma-treated PBS and concluded organic peroxides to be the responsible mediators. Boxhammer *et al.* investigated the mutagenic potential of a plasma device using surface microdischarge technology applied at bactericidal doses and did not observe increased mutation frequencies at the *hprt* locus of V79 Chinese hamster cells^44^. As pointed out by the authors, every atmospheric cold plasma device creates different plasma compositions and needs to be assessed for efficacy and safety.

The reader is referred to Moiseev *et al.*^45^ for a comprehensive diagnostic of the plasma discharge and the resultant active species generated using the system employed in the current study. While plasma exposure as used in the aforementioned study is not comparable to the exposure to plasma-activated solutions, any plasma treatment will result in the generation of some activated solutions, as cells are surrounded by even minimal liquid volumes. The exposure of cells over extended periods to plasma-treated solutions treated at high voltages and time frames of up to 20 min represents excessive treatment regimens. However, in terms of determining potential long-term harmful effects of repeated or even routine exposure to such activated solutions as would be the case in proposed applications such as antibacterial hand washes or disinfectants for the food industry, exposure to representative extreme albeit excessive conditions is warranted. Such considerations are even more pertinent where plasma-activated solutions have been suggested as intra-peritoneal washes^11^ to combat metastasis in cancer, where mutagenic compounds could further accelerate malignant progression of pre-cancerous cells.

## Conclusions

The use of atmospheric cold plasma or in fact plasma-activated solutions in medical applications requires not only adequate performance in terms of microbial inactivation, blood clotting, cancer inhibition or tissue regeneration efficiencies but also a safe operating window with regards to cytotoxic effects on exposed or surrounding tissues. Results obtained using HeLa and CHO-K1 cell lines as model cell systems showed that the cytotoxic effect of plasma treatment on cells is highly dependent on the surrounding milieu, with complete medium and serum providing protective effects over simple saline solutions and the presence of anti-oxidants such as pyruvate scavenging cytotoxic molecules such as hydrogen peroxide. Cytotoxic activity was, however, also retained in both simple and complex solutions post-treatment and showed subsequent adverse effects on untreated cells. Furthermore, long-term exposure of up to 6 weeks indicated an increased rate of mutagenic events in CHO-K1 cells cultured in the presence of plasma-treated solutions. Cytotoxic effects of plasma and in particular plasma-activated solutions which may retain their activity over extended storage periods may open up novel approaches in cancer treatment, especially as higher sensitivity of cancerous versus normal cells to these effects have been suggested in some studies. However, they also warrant further investigations to establish longterm safety for target and surrounding tissues in applications such as cancer treatment, wound healing or anti-microbial applications. Furthermore the exposure of tissue such as skin or wounds to plasma discharge will always also result in the generation of plasma-activated bio-fluids which must be considered with regard to the retention of plasma-activated species and their long-term effects.

## Materials and Methods

Unless specified otherwise all reagents were purchased from Sigma-Aldrich, Arklow, Ireland.

### Cell culture

The human cervical carcinoma cell line HeLa and the Chinese hamster cell line CHO-K1 were used for cytotoxicity and/or mutagenicity studies. HeLa cells were grown in Dulbecco’s Modified Eagle’s Medium (DMEM) supplemented with 2 mM L-glutamine and 10% foetal bovine serum (FBS). DMEM was either high glucose DMEM containing 4.5 g/l glucose and no pyruvate (Sigma-Aldrich D5796) or low glucose (1 g/l) and 0.11 g/l pyruvate (Sigma-Aldrich D5546). Culture medium for CHO-K1 consisted of DMEM/F12 (Sigma-Aldrich D6421) with 2 mM L-glutamine and 10% FBS. Cells were grown at 37 °C and 5% CO_2_ in a humidified incubator. Cells were detached using trypsin/EDTA and cell concentrations and viability were assessed using trypan blue counting.

Cell adhesion was assessed by crystal violet staining of trypsinized cells seeded at 1 × 10^6 ^cells/ml, left to re-adhere to 96-well tissue culture dishes for 1 day. Cell growth was assessed by crystal violet staining of trypsinized cells seeded at 2.5 × 10^4 ^cells/ml, left to re-adhere and grow for 3 days. Crystal violet staining: Culture supernatant was removed and cells were fixed with 70% methanol for 1 min followed by staining with 0.2% crystal violet solution for 10 min. After extensive washes with water, plates were air-dried and the crystal violet was solubilized with 10% acetic acid and absorbance measured at 560–600 nm on a spectrophotometric microplate reader (Biotek, Winooski, USA). Cell adhesion or growth were expressed as percentage of control cells.

### Plasma system

Plasma treatment was performed using a high-voltage di-electric barrier discharge atmospheric cold plasma system custom built at Dublin Institute of Technology (Dublin, Ireland) with a maximum output of 120 kV_RMS_ at 50 Hz which has been described in detail^45^,^46^.

### Plasma treatment

Trypsinized cells or medium in Eppendorf tubes were subjected to direct plasma exposure inside a rigid polypropylene container using a wide gap dielectric barrier discharge at 70 kV_RMS_ in air without post-treatment storage. For generation of plasma activated PBS and FBS, solutions were placed in the discharge area in 6-well plates or petri-dishes inside a polypropylene container and sealed with air-tight film. Plasma was generated at 70–80 kV_RMS_ in air, solutions were subjected to 24 hour post treatment storage time before opening.

### HPRT assay

The hypoxanthine phosphoribosyl transferase (HPRT) assay using CHO-K1 cells was employed to determine the potential of plasma activated FBS or PBS to induce mutagenic effects. In brief, cells were cultured in T25 flasks or 6-well plates in DMEM/F12 medium supplemented with 10% of FBS either untreated or treated at 80 kV_RMS_ DBD-ACP for 1, 5 or 10 min with 24 hour post-treatment storage time (experiment A). In experiment B the effects of treated PBS and treated FBS were compared. DMEM/F12 was supplemented with 10% of PBS treated at 80 kV_RMS_ for 0, 1, 5, 10, 15 or 20 min with 24 hour post-treatment storage time and 10% untreated FBS or vice versa. Cells were passaged every 3-4 days through trypsinization and reseeding at 2.5 × 10^4 ^cells/ml into fresh culture flasks. At the time of reseeding, cells were also plated at 1 × 10^4 ^cells/ml in round dishes (60 mm diameter) containing DMEM/F12 with 10% untreated FBS and 10 μg/ml 6-thioguanine (6-TG) as selection agent. Colony formation was determined after 10–14 days of incubation at 37 °C and 5% CO_2_ by staining with crystal violet. Plates showing no cell growth were re-assessed at day 18. As cells and cell clumps easily detach from colonies to re-attach and initiate further colonies in other parts of the plate, colony numbers did not represent a reliable estimate of mutation frequency. Instead plates were scored as HPRT+ or HPRT- based on presence/absence of colonies, respectively. Under the culture conditions employed, control plates were negative for colony formation at the start of the 40 day exposure period whereas ethyl methanesulfonate (EMS) was used as a positive control to induce colony formation. Cultures were performed as 3 independent replicates for each plasma-treated solution and triplicate 6-TG selection plates were set up from each of these replicates. In experiment A the independently treated solutions were pooled for each treatment time while they were kept separate in experiment B to account for potential variations between plasma exposures.

### Determination of hydrogen peroxide concentrations

Peroxide concentrations in solution were determined using oxidation of potassium iodide to iodine and spectrophotometric measurement at 390 nm. In brief, 50 μl of phosphate buffer and 100 μl of 1 M KI solution were added to 50 μl of plasma-treated PBS. After 30 min incubation, absorbance was read on a spectrophotometric plate reader at 390 nm. A standard curve of known hydrogen peroxide concentrations was included on each plate and used to convert absorbances into peroxide concentrations.

### Statistical analysis

Results are presented as means with standard deviations and statistical analysis where applicable was performed by analysis of variance (ANOVA) using GraphPad Prism (GraphPad Software Inc., La Jolla, USA).

## Additional Information

**How to cite this article**: Boehm, D. *et al.* Cytotoxic and mutagenic potential of solutions exposed to cold atmospheric plasma. *Sci. Rep.* **6**, 21464; doi: 10.1038/srep21464 (2016).

## Figures and Tables

**Table 1:** Occurrence of HPRT+ colonies in cultures supplemented with 10% FBS exposed to DBD-ACP for 0, 1, 5 or 10 min over a 39-day cultivation period (Experiment A).

FBS	Day in culture										
		0	3	8	11	15	18	22	29	32	39
Control	A	−	−	−	−	−	−	−	−	−	+
	B	−	−	−	−	−	−	−	−	−	−
	C	−	nd	−	−	−	−	+	−	−	+
1 min	A	nd	−	−	−	−	−	−	−	+	+
	B	nd	−	+	−	−	−	+	−	+	−
	C	nd	+	−	−	−	−	+	−	−	+
5 min	A	nd	−	−	+	−	−	+	−	−	+
	B	nd	+	−	−	+	−	−	−	−	−
	C	nd	−	−	−	+	−	+	−	+	+
10 min	A	nd	−	−	−	−	−	−	−	−	+
	B	nd	−	−	−	−	−	−	−	−	+
	C	nd	−	−	−	−	−	−	−	−	−

*minus* indicates no colonies were observed; *plus* indicates at least one of triplicate plates showed colonies; nd: not determined.

**Table 2:** Occurrence of HPRT+ colonies in cultures supplemented with 10% PBS exposed to DBD-ACP for 0, 1, 5, 10, 15 or 20 min over 43-day cultivation period (Experiment B).

PBS	Day in culture							
		0	8	15	22	33	40	43
Control	A	−	− − −	− − −	− − −	/	− − −	− − −
	B	−	− − −	− − −	− − −	− −/	+ − +	− − +
	C	−	− − −	− − −	− − −	− − −	− − −	− − −
1 min	A	nd	− − −	− − −	+ +/	− + −	− − −	− − −
	B	nd	− − −	− − −	− − −	− − −	− − −	/
	C	nd	−/−	− − −	− − −	− −/	− − −	− − −
5 min	A	nd	− − −	− −/	−//	+ + +	/	− + −
	B	nd	− − −	− − −	− − −	− − −	+ + −	− − +
	C	nd	− − −	− − −	− − −	− +/	+ − +	− − −
10 min	A	nd	− − −	/− −	− − −	− − −	− − −	− − −
	B	nd	− − −	− − −	− − −	− − −	− − −	+ + −
	C	nd	− − −	−//	+ + +	+ + +	+ − +	− + +
15 min	A	nd	− − −	+ + +	/− −	− − −	− − +	− + −
	B	nd	− − −	− − −	− − −	− − −	− − −	− −/
	C	nd	− − −	− − −	− −/	− − −	− − −	//−
20 min	A	nd	− − −	− − −	+ − +	− − −	− − −	− − −
	B	nd	− − −	− − −	− − −	− − −	− − −	− − −
	C	nd	− − −	− + +	− −/	− − −	− − −	−/−

*minus* indicates no colonies were observed; *plus* indicates plate showing colonies; nd: not determined.

**Table 3:** Occurrence of HPRT+ colonies in cultures supplemented with 10% FBS exposed to DBD-ACP for 0, 1, 5, 10, 15 or 20 min over 43-day cultivation period (Experiment B).

FBS	Day in culture							
		0	8	15	22	33	40	43
Control	A	−	−/−	− + −	− − −	− + +	/+ +	− − −
	B	−	− − −	− − −	− − −	//−	− − −	− − −
	C	−	− − −	+ +/	− − −	− + −	− − −	− − −
1 min								
	A	nd	/− −	− − −	− − −	+ + +	+ + +	+ + +
	B	nd	− − −	− − −	− − −	− − −	− − −	− − −
	C	nd	−/−	− − −	−//	− −/	− − −	− − −
5 min	A	nd	− − −	− − −	− − −	− − −	− − −	− − −
	B	nd	− − −	/	− − −	− − −	− − −	− − −
	C	nd	− − −	− − −	/−/	−//	+ + −	+ − −
10 min	A	nd	+ − +	/	− − −	− − −	− − −	+ + +
	B	nd	− −/	/	− − −	− − −	− − −	− − −
	C	nd	− −/	/	− − −	− − −	− − −	− − −
15 min	A	nd	− − −	− − −	− − −	− − −	− − −	−/−
	B	nd	− − −	− − −	+ + +	+ + +	+ − +	− − −
	C	nd	− − −	− −/	− − −	+ +/	− + −	+ + +
20 min	A	nd	−/−	− − −	− − −	− − −	− − −	− − −
	B	nd	− + −	− − −	− − −	− − −	− − −	− − −
	C	nd	− − −	− − −	− − −	− −/	/− −	− − −

*minus* indicates no colonies were observed; *plus* indicates plate showing colonies; nd: not determined.

**Table 4:** Occurrence of HPRT+ colonies in cultures supplemented with H_2_O_2_ between 0 and 4 × 10^−4^% (w/v) over 40-day cultivation period.

H_2_O_2_ [%]	Day in culture							
		0	6	13	20	27	33	40
Control 0	A	−	− − −	− − −	− + +	+ + −	+ − −	− − +
	B	−	− − −	− − −	− − +	− − −	− − −	+ − −
	C	−	− − −	− − −	+ − +	− − −	− − −	− − −
2 × 10^−4^ (65 μM)	A	nd	− − −	+ + +	+ − +	+ + +	− − −	+ + +
	B	nd	− − −	+ + +	+ − −	+ + +	− − +	+ + −
	C	nd	+ − −	− − −	− − +	+ + −	− − −	+ − −
2.5 × 10^−4^ (82 μM)	A	nd	− − −	− − −	− − −	+ + +	+ + +	− + +
	B	nd	+ − −	− − −	− − −	+ + −	+ + +	+ + +
	C	nd	+ − −	− − +	− − +	− − −	− + −	+ + +
3 × 10^−4^ (98 μM)	A	nd	− + −	− − −	− − −	− − −	− − −	+ − −
	B	nd	+ − −	− − +	− − −	− − −	− − +	− − −
	C	nd	− − −	− − −	− − −	+ − +	− − −	− − +
4 × 10^−4^ (130 μM)	A	nd	− − −	− + +	+ + +	− − +	− + +	+ + +
	B	nd	− + −	/+ +	+ + +	− − −	+ − +	+ + +
	C	nd	− − −	+ + +	+ + +	+ + −	− − −	− + +

*minus* indicates no colonies were observed; *plus* indicates plate showing colonies; nd: not determined.
